# Linking key husbandry factors to the intrinsic quality of broiler meat

**DOI:** 10.1016/j.psj.2022.102384

**Published:** 2022-12-06

**Authors:** Joanna Marchewka, Patryk Sztandarski, Magdalena Solka, Helen Louton, Katharina Rath, Lukas Vogt, Elke Rauch, Dionne Ruijter, Ingrid C. de Jong, Jarosław O. Horbańczuk

**Affiliations:** ⁎ Institute of Genetics and Animal Biotechnology, Polish Academy of Sciences, Jastrzebiec, 05-552 Magdalenka, Poland; ⁎⁎ Animal Health and Animal Welfare, Faculty of Agricultural and Environmental Sciences, University of Rostock, 18059 Rostock, Germany; † Quality assurance animal welfare, Naturland – Association for Organic Agriculture e.V., 82166 Graefelfing, Germany; # Chair of Animal Welfare, Ethology, Animal Hygiene and Animal Husbandry, Department of Veterinary Sciences, Faculty of Veterinary Medicine, 80539 Munich, Germany; ‡ Wageningen Livestock Research, Wageningen University & Research, 6700 AH Wageningen, The Netherlands

**Keywords:** broiler, genetics, diet, stocking density, enrichment

## Abstract

Broiler farming is the fastest-growing animal production sector and broiler meat is the second most-consumed meat in the world. The intensification of broiler production often has a negative impact on the meat quality and carcass characteristics. Consumers, however, expect a quality product from animals reared extensively on farms providing good animal welfare, often intuitively associated with extensive farming practices. Therefore, this literature review investigates how the critical factors contributing to the degree of extensiveness of broiler production affect the quality of meat. We used the data from scientific articles published in the years 2012–2021 to analyze the effect of diet (n = 409), genetics (n = 86), enrichment (n = 25), and stocking density (n = 20) on meat quality and carcass characteristics. Minerals and microelements supplementation in the diet improved all the meat quality aspects: sensory, physical, and chemical in most studies. Minerals and enzymes in the diet had beneficial effects on carcass characteristics, unlike feed restriction and ingredient substitutions. The impact of outdoor access on meat quality and carcass characteristics was most frequently examined, in contrast to the use of perches or effects of litter quality. Overall, enrichment did not affect the meat's sensory or physical parameters, but outdoor access improved its lipid composition. Lower stocking density deteriorated intramuscular fat content, decreased tenderness and juiciness, yet lowered cooking and drip loss, and increased carcass and breast muscle yields. When it comes to genetics, in general, slow growing broiler strains have better meat quality parameters, especially regarding yellowness (b*), redness (a*), cooking and drip loss. Our review shows that the factors which contribute to extensiveness of broiler production systems and birds’ welfare also affect meat quality and the carcass characteristics.

## INTRODUCTION

Meat is the most important source of animal protein for the human diet (McAfee et al., 2010; Lawrie and Ledward, 2014). However, despite the continuous growth in its consumption, the European Union's Agricultural Outlook predicts a turning point in the trend caused by significant changes in human dietary patterns (EU Commission, 2019). Consumers increasingly pay attention to the quality, safety, authenticity, key welfare associated husbandry factors and credence attributes of meat products (Augère-Granier, 2019).

The assessment of meat quality relates to its intrinsic qualities and extrinsic factors. The former describes the product characteristics, and the latter refers to production system characteristics like rearing conditions, environmental impact, or price (Hocquette et al., 2014). Consumers, especially from western countries and EU, believe that low-input, extensive systems are more sustainable, superior for the birds’ welfare (Erian and Phillips, 2017) and provide better product quality (Mulder and Zomer, 2017). That explains their aversion toward livestock species such as broilers reared specifically for their fast growth (Cavani et al., 2009) and kept at relatively high stocking densities (Augère-Granier, 2019). However, the degree of the extensiveness of a production system (conventional, organic, free-range) is often confounded with various aspects like the utilized breed, applied stocking density or availability of the outdoor range access.

Meat quality is often valued by its industrial characteristics. So, in the quest for more homogeneous products, the integrated quality concept includes all important intrinsic properties like color, pH, water holding capacity (**WHC**), cooking losses, tenderness, chemical composition, or fatty acid content and key husbandry factors. WHC and drip loss are strongly correlated with pH value. As pH declines, the meat becomes paler, softer, and higher in drip loss. The color parameter is usually expressed by the lightness (L*), redness (a*) and yellowness (b*) values within the color space system (Mir et al., 2017a). In meat classification, the higher the L*value, the paler the meat. A high and positive a* value in meat classification means an intensive red coloring, while a high and positive b* value indicates the undesirable intensive yellowness. High drip loss is not only visually unattractive to consumers, but it can also result in excessive cooking losses and the dryness of meat after cooking. Meat-solidity is another vital quality characteristic for meat processing, measured by meat texture (i.e., its reaction to shear or compression force) and the performance at distortion.

Carcass composition defines the relative proportions of dissected skeletal muscle, adipose tissue, and bone measured in growth experiments (Mir et al., 2017a). Carcass measurements of breast and drumstick and carcass weight are frequently used to classify carcasses for pricing and marketing activities and to estimate total carcass skeletal muscle mass of meat chickens. Even though consumers value broiler meat in terms of product quality (i.e., tenderness, fat content, color etc.), broiler producers’ revenue is directly related to carcass characteristics (i.e., carcass weight, fat distribution). For instance, weight gain and feed conversion ratio (**FCR**) influence profitability (da Silva et al., 2017). Thus, carcass characteristics are essential for the success of the whole broiler meat production chain as meat quality (Tijare et al., 2016).

Both broilers’ meat quality parameters and carcass characteristics are strongly linked to the key husbandry factors typical for a certain production system. For instance, the fatty acid profile of meat reflects that of the diet used in a certain production system (Jaturasitha et al., 2008). Furthermore, diet density affects meat yield and quality, but these effects differ depending on the genotype of broilers used in a certain system (Wang et al., 2013).

As for the environment, broilers are predominantly housed in conventional barren-floor housing systems where environmental enrichment could promote their activity and welfare (Riber et al., 2018). Some meat quality parameters, for instance, juiciness, were attributed to a difference in activity of the broilers, motivated by the environmental enrichments (Riber et al., 2018). Thus, intensive versus extensive farming may lead to differences in meat texture, where the more active birds may produce tougher meat (i.e., increased shear force readings). Furthermore, since stocking density affects the activity level, welfare, and product quality (Nasr et al., 2021), lower stocking densities in extensive systems may affect meat quality and carcass yield.

Genotype can also strongly affect several parameters like postmortem pH decline, color, drip loss and cooking yield (Rama Rao et al., 2003). Also, an early slaughter age in broilers has an impact on the meats’ chemical parameters and taste (da Silva et al., 2017). Slower-growing genotypes are used in more extensive production. Their feed efficiency is lower than in fast-growing broilers, it takes longer for them to reach market weight, but the meat quality issues typical for intensive systems are reported to be less. For example, the breast muscle of slow growing birds had more glycogen reserves at slaughter than that of fast-growing chickens. Moreover, the pH decline of slower-growing chicken lines was faster than in fast-growing ones (Berri, 2000). Effects of genotype were also observed on breast meat composition, quality, sensory aspects, and shear force (Lonergan et al., 2003). As for the technological quality of meat, genotype strongly affects physicochemical and sensory parameters (Roy et al., 2007).

In this literature review, an overview is presented of how key broiler husbandry factors of diet, genotype, quantity, and quality of space have been linked to chicken meat quality and carcass characteristics across various production systems in the studies performed in the last decade.

## METHODOLOGY

### Intrinsic Meat Characteristics and Key Husbandry Factors

**Figure 1:**
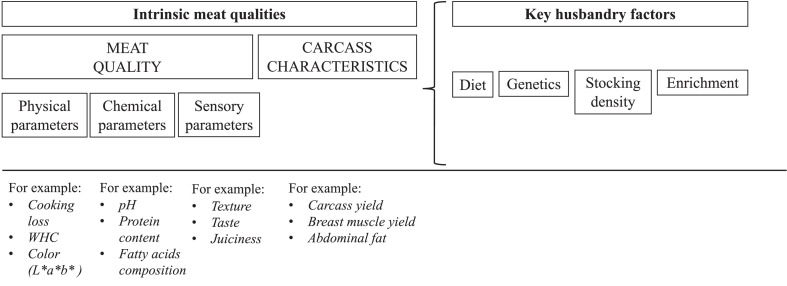
Intrinsic meat characteristics and key husbandry factors for which the literature (2012–2021) was reviewed.

The concept of food quality is not universal and depends on who is making the definition (Becker, 2000). The current review defined intrinsic meat characteristics as meat quality, further divided into physical, chemical, and sensory parameters and carcass characteristics. As presented in Figure 1, we considered the following critical husbandry factors: diet, genetics, stocking density, and enrichments.

### Data Collection

This was a semistructured review, as the subject it dealt with was multidisciplinary and broad, which would hinder a full systematic review process. It was based on the relevant scientific literature from the Web of Science database. As the body of the literature identified was very broad, to homogenize the search and avoid articles repetition this single database was used. Keyword queries for each of the 4 factors: diet, stocking density, environmental enrichment and genetics were developed based on expert consultations to best cover the literature. Queries regarding broiler meat and carcass quality were uniform across factors, while the specific keywords describing each factor were unique (see supplementary material 1 for each query). All the searches were limited to the keywords in the titles of the articles. Documents in the review were published in the years 2012–2021. Articles which described local, native, and commercially unavailable breeds or genetic lines of chickens were not included.

### Articles Grouping and Presentation of Results

Table 1 presents the search outcome for each key husbandry factor in terms of: the number of identified articles, the number of articles included in this review and the number of excluded ones.

**Table 1:** Numbers of identified articles for each broiler husbandry factor.

Key broiler husbandry factor	Identified articles (n)	Relevant articles (n)	Excluded articles (n)
Diet	705	409	296
Enrichment	90	25	65
Stocking density	26	20	6
Genetics	204	86	118

The information for each key husbandry factor is presented as either concerning the meat quality or carcass characteristics. The authors of the current review did not set own thresholds for the optimal levels of each trait. The results have been presented as interpreted in the reviewed texts (improvement/deterioration or no effect). The reference level to the treatment group is, therefore, in most cases, the control group. In the studies where such interpretation of the results was not available, the exact levels of the investigated parameter were reported. The results for the factor ‘diet’ were presented differently than for other key husbandry factors since this information was most extensive.

#### Diet

As there were many papers on the factor diet (n = 409), they were subdivided into fourteen topic groups: plant extracts, enzymes, microelements, vitamins, minerals, probiotics/prebiotics, protein alternatives, antibiotic alternatives, ingredients substitution, feed form, feed restriction, feed alternatives, pasture diet/roughage, organic production. The grouping reflects the most recent trends in broiler nutrition publications that emerged from the reviewed abstracts. Subsequently, the treatment effects were presented in the figures as proportion (%) of studies showing an improvement/deterioration or no effect for each parameter group (meat quality: physical, chemical and sensory or carcass characteristics), separately, while in the text we cited only studies that we found most interesting.

#### Enrichment, stocking density, and genetics

The entire information for enrichment, stocking density, and genetics was presented regarding the levels of the treatments for each of the key husbandry factors.

The studies investigating genetics as the key husbandry factor to influence meat quality juxtaposed at least 2 breeds. When compared, one breed has automatically higher values than the other. To avoid duplications, the increased values were counted, while the reference breed was only mentioned. Chickens in the current literature review were divided into the categories in accordance with their growth rates: fast growing chickens with daily weight gain (DWG) of >35 g, medium growing birds with DWG of 20 to 35 g and slow growing birds with DWG of <20 g (Dal Bosco et al., 2012). Moreover, we have distinguished dual purpose type of birds, according to the definition as described by Tůmová et al. (2021).

## RESULTS

### Diet and the Meat Quality

All of the 409 reviewed articles touched upon the meat quality aspects.

The reviewed authors reported improved meat quality parameters in the studies where minerals (n = 14 studies) and microelements (n = 38 studies) were applied. The enhanced quality of the produced meat, mainly organoleptic parameters and the level of omega-3 fatty acids, by using zeolite were described (Malek et al., 2012). Banaszak et al. (2021) provided an insight into aluminosilicates with the advantages of zeolite and halloysite in particular on the content of protein and on the WHC. Zinc in organic form and zinc in combination with amino acids reduced drip loss (Sałek et al., 2020), increased the meat moisture, protein content and pH, improved the b*, juiciness, tenderness, and taste of the meat, while the lipid content of the meat was significantly lower in the treatment groups (Abd El-Hack et al., 2021). Benefits of iron supplementation were mainly: increased L* value and b* value, iron and copper content in the breast muscle and increase in the a* of the breast meat and its pH at 24-h post mortem (Behroozlak et al. 2021)*.*

Probiotics and prebiotics were described to affect positively the physical parameters of the meat in 42% of the articles (n = 32 articles) and chemical parameters in 55%. Zheng et al. (2014) reported the benefits of *Enterococcus faecium*: intensified the meat color, increased the WHC and pH of the pectoralis muscle. The microorganism-based probiotic from the cattle rumen and chicken intestine containing *Lactobacillus spp., Biofidobacterium spp*., *Streptooccus spp.,* and *Bacillus spp.* increased protein content and decreased fat (Suryadi et al., 2019). *B. amyloliquefaciems* increased the content and concentration of selected fatty acids and improved the ratio of PUFA to saturated fatty acids (Wei et al., 2017). Adding *Bacillus pumilus* to the diet improved oxidative stability in meat (Bonos et al., 2021). Supplementation with up to 2% of fermented pomegranate (*Punica granatum)* improved nutritional value and shelf life of meat (Ahmed et al., 2017).

Adding enzymes to the diet (n = 12 studies), diet under organic production (n = 4 studies), or when feeding pasture/roughage (n = 6 studies) brought about only a few beneficial effects mainly on the physical and chemical parameters of the meat. Shoaib et al. (2021) described the positive influence of lipase (alone or in combination with bile acid) on meat quality, mainly WHC. The proportion of PUFAs, n-6 and n-3 fatty acids and the PUFA/SFA ratio were improved in canola-based diets containing potassium humate and enzymes (Disetlhe et al., 2019). The dietary enzyme inclusion increased the crude protein (CP) content and reduced the fat content of the meat, as well as increased its WHC together with a reduction of the cooking loss rate (Abdel-Daim et al., 2020).

Access to pasture diet combined with concentrate feed restriction elicited remarkable changes in the bird's antioxidant system, while the changes in the meat's oxidative stability were less pronounced and more difficult to interpret (Michiels et al., 2014). Birds foraging on pasture showed a darker and more intense yellow color of their breast meat as compared with those fed concentrate feed delivered indoors, while the muscle total fatty acid content was also higher in the outdoor reared birds (Michiels et al., 2014). Adding organic grass-clover, as a typical component of pasture diet, to the birds’ feed increased the meat's alpha-linolenic acid (C18:3*n*-3) content. A lowered tocopherol content in the meat from broilers fed with increased grass-clover protein demonstrated the need for higher amounts of antioxidants due to the high content of unsaturated fat (Stødkilde et al., 2020). The breast meat of chickens with access to pasture diet was found to have a higher protein content, while the meat's color was related to the ultimate pH, which was observed to be significantly higher in free-range chickens (Funaro et al., 2014). Dietary supplementation with fresh chicory forage increased the total amino acids and amino acids in muscles and improved muscle nutritional value and flavor (Zheng et al., 2019).

Regarding deterioration of meat quality, studies on different dietary aspects suggest effects on sensory, physical and chemical properties. Examples are feed restriction (n = 25 studies; 40%, 25%, and 33%, respectively), antibiotic alternatives (i.e., herbal blends, essentials oils, glycerol monolaurate, phenolic, or chitosan compounds) (n = 21 studies; 20%, 6% and 6%, respectively), plant extracts (n = 86 studies; 17%, 18%, and 10%, respectively), protein alternative (n = 52 studies; 10%, 9%, and 7%, respectively) and feed alternatives (n = 69 studies; 7%, 5%, and 2%, respectively).

Most recent research found that the diet containing 6% lower crude protein was associated with changes in the meat quality parameters including increased darkness, meat color intensity, drip loss, and muscle fiber area (Chodová et al., 2021). Feed restriction during the first growth period (from 13 to 21 d of age) tended to increase white-striped breasts (69.5 vs. 79.5%) with the females showing fewer wooden breasts than males (8.0 vs. 16.3%). The feed restriction by 30% negatively affected the concentrations of alpha and gamma-tocopherol and oxidative stability in the broiler meat, while the 20% feed restriction in chickens housed in mobile boxes significantly increased the *n*-3 fatty acids content and h/H index, reduced the *n*-6/*n*-3 ratio, atherogenic and thrombogenic index, which was beneficial for human health (Englmaierová et al., 2021).

Application of the antibiotic alternatives in broiler diet was reported to have few desirable results. Glycerol monolaurate (**GML**) brought positive effects to meat quality (Fortuoso et al., 2019), while negative effects were caused after applying candlenut kernel (Rasid et al., 2019), camelia oil and seeds (Ciurescu et al., 2016) or grape seed oil supplementation (Paula Turcu et al., 2021).

Alternative protein sources brought about various negative effects when compared to control groups or other treatments. The investigated alternatives included: mealworm (Shaviklo et al., 2021), defatted larvae meal (*Hermetia illucens L.*) alone or in combination with spirulina Gkarane et al., 2020, Schiavone et al., 2019; full-fat larvae meal (*Hermetia illucens L.*) (Murawska et al., 2021); oleic peanut diet (Lipiński et al., 2019), *Boswella Serrata resin* (Kiczorowska et al., 2020).

Some feed alternatives, like broken rice and dried distillers grains with soluble (**DDGS**) in a flaxseed-based diet had a negative effect on the meat color and appearance, flavor, texture, juiciness, and overall acceptability of the meat (Mir et al., 2017b). Sugar syrup as a substitute for starch/grains and vegetable oil increased significantly the cooking loss of the breast meat and the cooked thigh meat had a harder texture (Hashim et al., 2013). Extruded flaxseed reduced the oxidative stability in the meat (Anjum et al., 2013).

**Figure 2:**
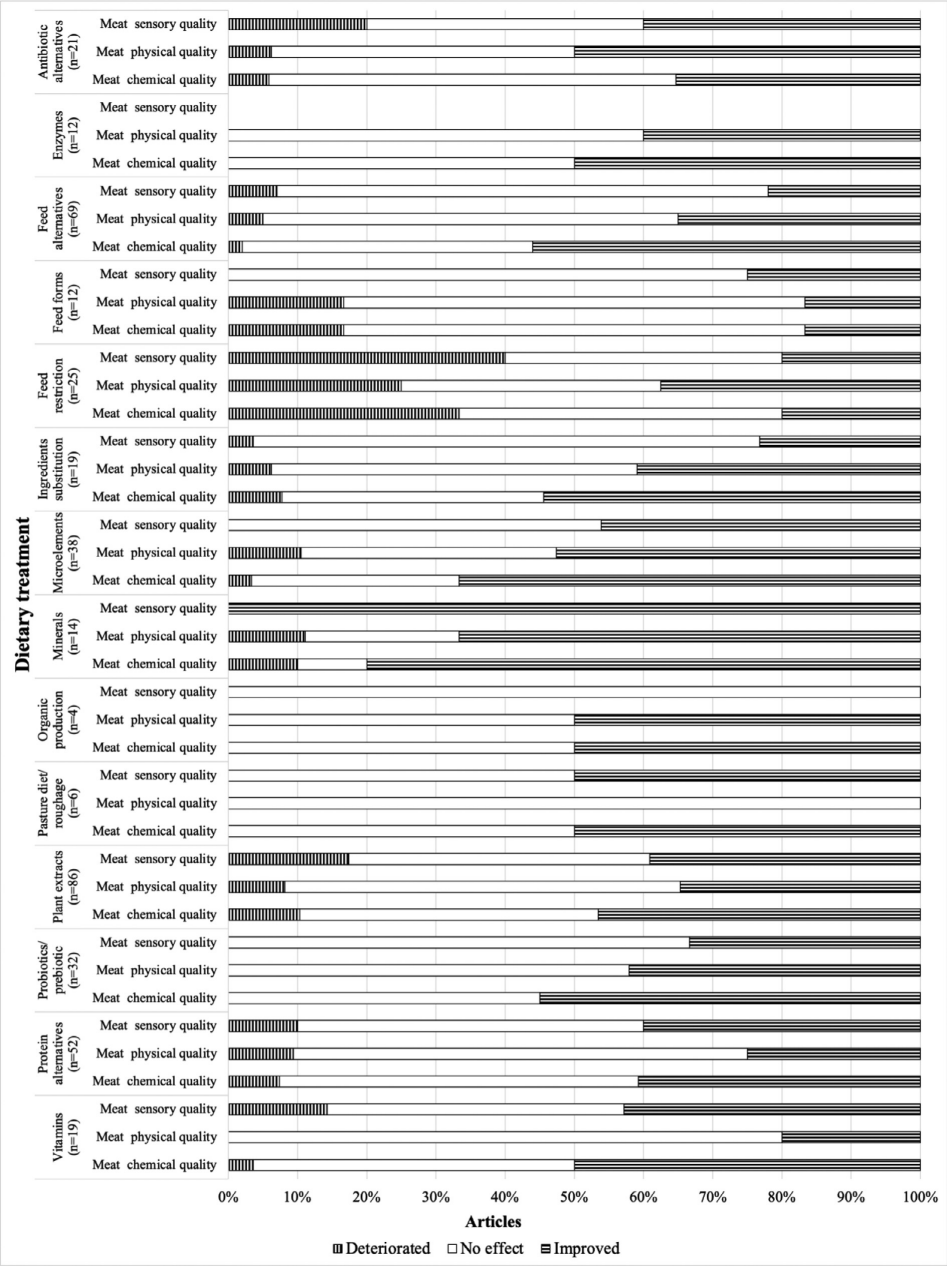
Overview of the proportions of the results concerning meat quality divided into meat sensory, physical and chemical quality, identified in each of 14 dietary treatments subtopic groups, where n = the number of the research articles falling under particular subtopic group.

Moreover, studies examining feed forms (n = 12 studies; 17% for physical and chemical characteristics), minerals (n = 14 studies; 11% and 10%, respectively), ingredient substitution (n = 88 studies; 8% and 13%, respectively) and microelements (n = 38 studies; 11% and 3%, respectively) found solely adverse effects on the physical and chemical parameters of broiler meat (Figure 2). The exception was the group of articles describing the effects of the administration of vitamins, where some negative effects were described only for the sensory parameters of meat, as found by 14% (n = 19 studies) of the identified literature (Figure 2).

### Enrichment and the Meat Quality

Overall, the review of the available literature showed that most research was carried out in relation to effects of outdoor access, followed by few studies on perches use effects, while no effects of other enrichments on meat quality were examined. Four studies analyzed the effect of providing perches on the chemical quality of the meat. None of the authors found any effect of perches on the pH in general, moisture, protein, or lipids content.

Regarding the chemical quality of the meat of broilers with outdoor access, different effects were observed. Two out of 10 studies observed a decline in meat pH as compared to indoor housing (da Silva et al., 2017; Zaid et al., 2020), while another two observed higher pH values in broilers with outdoor access (Chen et al., 2013; Stadig et al., 2016). Six studies did not see an effect of outdoor access on the meat pH (Kuttappan et al., 2013; Funaro et al., 2014; Michalczuk et al., 2017; Skřivanová et al., 2017; Mosca et al., 2018; Skomorucha et al., 2020; Sampels et al., 2021). Moisture, protein, fat content and lipids were considered in 6, 8, 8, and 10 studies respectively, showing inconsistent results. Two studies examining the moisture level observed an increase (Funaro et al., 2014; da Silva et al., 2017), one a decrease (Skřivanová et al., 2017) and four reported no effect at all (Stadig et al., 2016; Michalczuk et al., 2017; Evaris et al., 2021). An increase in the protein level was observed by 4 of the 8 authors who tested for this factor (Funaro et al., 2014; da Silva et al., 2017; Mosca et al., 2018; Evaris et al., 2021), the others did not observe any effect (Chen et al., 2013; Stadig et al., 2016; Michalczuk et al., 2017; Skřivanová et al., 2017). Effects on fat content also showed varying results. In 2 studies, a decrease was found (da Silva et al., 2017; Evaris et al., 2021), in 2 an increase (Chen et al., 2013; Mosca et al., 2018), the remaining 4 found no effect of outdoor access on the parameter (Stadig et al., 2016; Michalczuk et al., 2017; Skřivanová et al., 2017; Sampels et al., 2021). Most authors conclude that outdoor access has a positive effect on the lipid composition (Chen et al., 2013; Funaro et al., 2014; Stadig et al., 2016; Michalczuk et al., 2017; Evaris et al., 2021; Sampels et al., 2021), only one of the studies (da Silva et al., 2017) observed a negative effect and 3 no effect (Michiels et al., 2014; Skřivanová et al., 2017; Skomorucha et al., 2020).

Considering perches, no effect was observed on the physical meat quality such as WHC (Kiyma et al., 2016; Fidan et al., 2020), cooking loss (Fidan et al., 2020), or drip loss (Kiyma et al., 2016). None of the studies assessed effects of perches on the occurrence of thawing loss, shear force or white striping.

In contrast, outdoor access did have an effect on the physical meat quality. Two of 6 studies observed increased WHC with outdoor access (Funaro et al., 2014; Mosca et al., 2018), one a declining effect (da Silva et al., 2017), and 3 no effect (Michalczuk et al., 2017; Skomorucha et al., 2020; Sampels et al., 2021). A reduction in cooking loss was observed by Funaro et al. (2014). Chen et al. (2013), Stadig et al. (2016), Michalczuk et al. (2017), Mosca et al. (2018), Zaid et al. (2020) and Sampels et al. (2021) did not observe any effect. Drip loss was predominantly not affected by outdoor access (Chen et al., 2013; Zaid et al., 2020; Sampels et al., 2021); only 2 studies observed a reduction (Funaro et al., 2014; Stadig et al., 2016) and one an increase (Skomorucha et al., 2020). Thawing loss was only assessed by two studies and was not affected by outdoor access (Michiels et al., 2014; Sampels et al., 2021). Considering shear force, the authors predominantly concluded that outdoor access increased the shear force (Fan et al., 2013; Funaro et al., 2014; da Silva et al., 2017; Michalczuk et al., 2017). Stadig et al. (2016) and Samples et al. (2021) did not observe any effect and Skrivanova et al. (2017) observed a reduction.

The impact of the range use access, as an environmental enrichment on the sensory quality of the meat was assessed in one study and did not report any effect (da Silva et al., 2017).

Aksit et al. (2017) and Fidan et al. (2020) observed a reduction on the L* when perches or cooled perches were provided. Contradicting results were stated by the authors who assessed the extrinsic factor ‘outdoor access’ on L* as a sensory meat quality parameter. Three of 8 studies evaluating this parameter observed an increase in L* (Chen et al., 2013; Stadig et al., 2016; Michalczuk et al., 2017), 2 a decrease (Funaro et al., 2014; Michiels et al., 2014), and 3 no effect (Skomorucha et al., 2020; Zaid et al., 2020; Sampels et al., 2021). Contradicting results were observed considering the effects of perches on b*. Aksit et al. (2017) observed an increase, Kiyma et al. (2016) a decrease. An effect of perch cooling (Fidan et al., 2020) or light (Aksit et al., 2017) on b* was not observed. Considering outdoor access, 3 of 7 studies observed an increase of b* (Funaro et al., 2014; Michiels et al., 2014; Stadig et al., 2016; Michalczuk et al., (2017) observed the decline of b* and 3 authors did not observe any effect (Skomorucha et al., 2020; Zaid et al., 2020; Sampels et al., 2021). Contradicting results were also observed in the assessment how perches, light or litter affected the sensory meat quality of a*. Kiyma et al. (2016) observed the decline of a* with perch availability for all the factors. Aksit et al. (2017) observed an increase in a* upon the provision of perches and Aksit et al. (2017) and Fidan et al. (2020) did not observe any effect of perch cooling, litter thickness, or light on a*.

The outdoor access did not seem to affect the a* of meat according to 5 studies (out of 6) (Stadig et al., 2016; Michalczuk et al., 2017; Skomorucha et al., 2020; Zaid et al., 2020; Sampels et al., 2021).

Taste, odor, and texture were only assessed in the studies which evaluated the effect of outdoor access. Predominantly no or a positive effect was observed. No study examined the effect of indoor enrichment such as light or perches on the taste, odor, or texture of the meat. Da Silva et al. (2017) and Zaid et al. (2020) observed a higher taste evaluation of broiler meat if the chickens had had outdoor access, others observed no effect on the taste (Stadig et al., 2016; Skřivanová et al., 2017). A negative effect on the taste was not observed. Stadig et al. (2016) reported a positive effect of outdoor access on the odor, and Skřivanová et al. (2017) no effect. Considering the texture, predominantly a positive effect (Stadig et al., 2016; da Silva et al., 2017) was observed, Chen et al. (2013) noticed no effect, while Zaid et al. (2020) reported a negative effect.

### Stocking Density and the Meat Quality

Stocking density is defined as the number of birds or kilogram of live weight reared per square meter (EU, 2007). There is a considerable distinction related to the stocking density of broiler among different countries; that is, the stocking density in Netherlands is 45 to 54 kg/m^2^, United Kingdom is 40 kg/m^2^, and Switzerland is 30 to 36 kg/m^2^ (Yuan, 2017). For this review, low stocking density was set as ≤10 birds/m^2^, medium stocking density as 11 to 16 birds/m^2^, and high stocking density as ≥17 birds/m^2^ to account for the variability in the thresholds among countries. Various studies reported an effect of stocking density on chemical, physical and sensory meat quality.

In general however, regardless of thresholds for high and medium stocking density a number of studies prove that lower stocking densities resulted in improved meat quality be it meat pH (Tong et al., 2012; Skrivanova et al., 2017; Nasr et al., 2021), or faster pH decline post slaughter (Wu et al., 2020a). Only one study reported a reversed trend and this concerned fat content (Simitzis et al., 2012).

The cooking and drip loss have been reported to be lower for a stocking density of 14 birds/m^2^ compared to a stocking density between 18 and 20 birds/m^2^, that is, medium stocking density vs. high stocking density in this review (Wu et al., 2020a; b; Nasr et al., 2021). Goo et al. (2019) reported a lower WHC for a stocking density of 9 birds/m^2^ compared to 18 birds/m^2^, low stocking density vs high stocking density in this review (Goo et al., 2019).

Goo et al. (2019) reported a higher L* of the meat color for a stocking density of 9 birds/m^2^ than for 18 birds/m^2^, which was considered a low stocking density vs high stocking density. On the other hand, Wu et al. (2020a) reported a lower L* for a stocking density of 14 birds/m^2^ than for 18 birds/m^2^, that is, medium stocking density vs. high stocking density. Lower acceptability was reported for a stocking density between 4.15 and 12 birds/m^2^ compared to a stocking density between 18 and 20 birds/m^2^, that is, low and medium stocking density vs. high stocking density (Skřivanová et al., 2017; Ebeid et al., 2019). Meat tenderness and juiciness can also be affected by stocking density. This is negatively correlated with intramuscular fat, which was lower for birds reared at a lower stocking density (Skřivanová et al., 2017).

### Genetics and the Meat Quality

Supplementary material 2 refers to the studies that described the effect of the genetic strain on broiler meat.

The factor genetics was defined in this review as the impact of particular broiler lines on the meat quality and carcass characteristics. Aspects like parts of the genome, SNPs or genes were excluded. The categorization into groups (fast vs. slow-growing) was based on the particular studies reported in this review.

Thirty-two studies analyzed effects of genetics on chemical parameters of meat quality. Six of them indicated a relationship between the bird type according to its growth rates and the meat's pH. Lower meat pH values were seen in dual-purpose chicken compared to fast-growing broilers (Siekmann et al., 2018b). Fast-growing broilers were characterized by lower meat pH and higher protein content, as compared to slow-growing ones (Koçer et al., 2018) and higher abdominal fat levels compared to medium and slow-growing broilers (Tůmová et al., 2021). In contrast, four papers showed higher protein content in slow-growing broilers than in fast-growing ones and one study in comparison to medium-growing ones. Six papers dealt with fat content. Lower fat content was seen in slow-growing broilers compared to fast-growing broilers (Pietrzak et al., 2013). The intramuscular fat content was higher in dual-purpose chicken than in slow-growing lines (Mueller et al., 2020). Slow-growing broilers showed higher amounts of SFA, stearic acids and PUFAs than medium and fast-growing ones (Dal Bosco et al., 2012; Boschetti et al., 2016). Contrastingly, one study reported lower total fatty acids and SFA in slow-growing broilers compared to fast-growing broilers (Sosnówka-Czajka et al., 2017). Higher content of PUFAs was observed in medium-growing broilers compared to fast-growing broilers (Boschetti et al., 2016), while compared to slow-growing broilers there was no significant difference (Boschetti et al., 2016). Eleven of the articles on the chemical parameters of meat quality revealed no significant differences between broilers' genetic lines.

Thirty-one articles studied the effects of broilers' genetics on the physical meat quality parameters. Four studies reported on WHC, 3 on cooking losses in fast-growing broilers. They showed variability of both parameters in those birds depending on their hybrid. One study showed higher cooking losses in dual-purpose chicken (Siekmann et al., 2018b), 3 in slow-growing (Pietrzak et al., 2013; Wen et al., 2017; Chodová et al., 2021), and one in medium-growing ones (Chodová et al., 2021). Results on drip loss were also inconsistent. In 2 studies, they were higher and in the other two lower for fast-growing broilers compared to slow-growing ones, as well as compared to medium-growing broilers in one study. Thawing losses were lower (Mueller et al., 2018) and shear force values were higher in fast-growing broilers than in dual-purpose, layer hens, medium, and slower-growing chickens (Mueller et al., 2018; Devatkal et al., 2019; Chodová et al., 2021; Sampels et al., 2021). For medium-growing broilers, one study reported that thawing losses were lower in slow-growing broilers and TETRA HB-Color broilers than in medium-growing ones in a particular farm (Almasi et al., 2015). Lower L* values were reported by the same study for TETRA HB Color broilers compared to Shaver Farm broilers (Almasi et al., 2015). Slow-growing broilers had higher shear force values in 3 studies. In thirteen studies no significant effects of genetics on physical meat quality parameters were observed.

Twenty-five papers investigated the influence of broiler genetics on sensory meat quality parameters. Among various fast-growing broiler hybrids, 4 studies found an effect of genetics on color. In slow-growing broiler lines variability between those broilers in L* (10 studies), a* (5 papers), and b* values (4 studies) was reported. Slow-growing broilers were reported to have better taste and tougher texture of meat, as compared to fast-growing ones (Pellattiero et al., 2020). Fourteen papers found no significant effect of genetic influence on the sensory meat quality parameters.

### Diet and Carcass Characteristics

**Figure 3:**
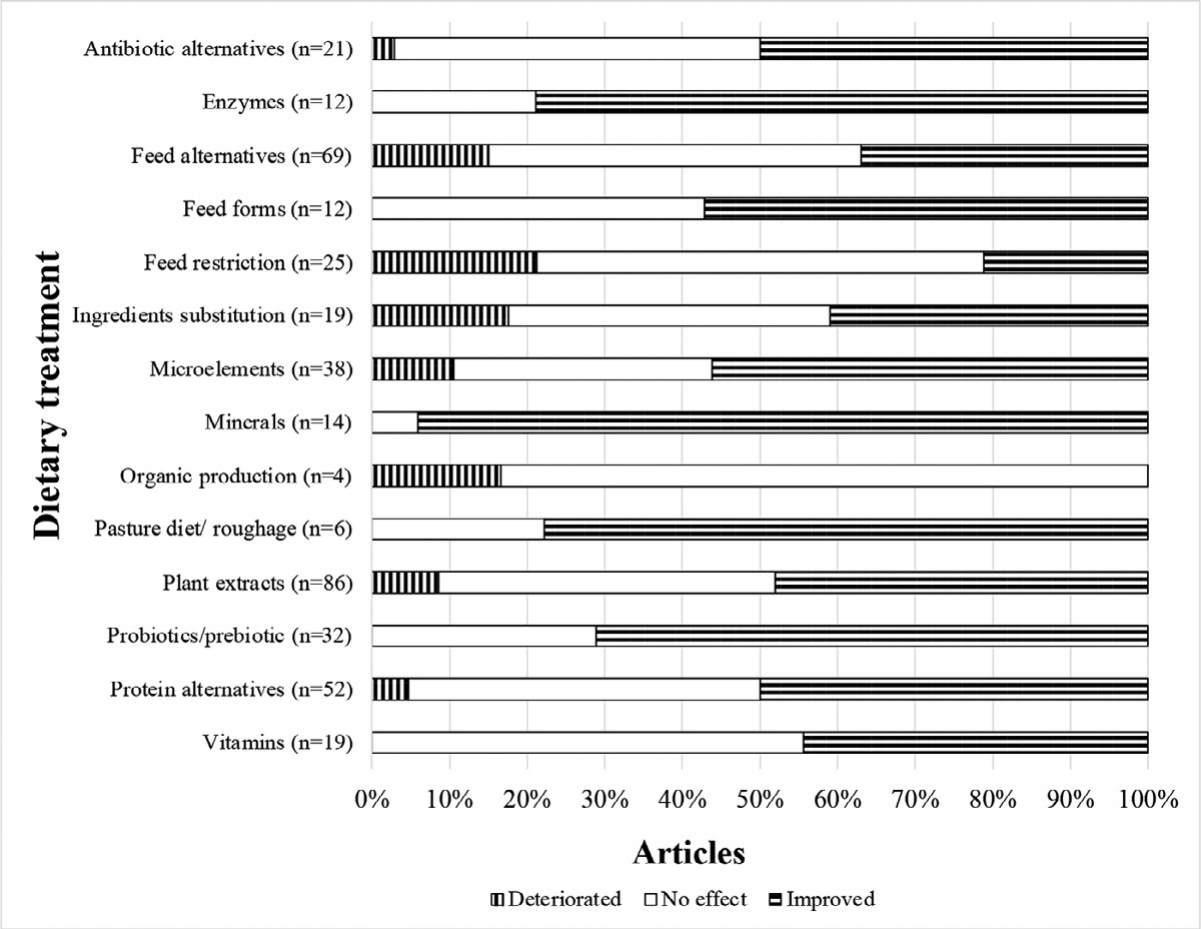
Overview of the proportions of the results concerning carcass characteristics identified in each of 14 dietary treatments subtopic groups, where n = the number of the research articles falling under particular subtopic group.

Further reviewed literature focused on the dietary effects on carcass characteristics. The distribution of the identified results is presented in Figure 3.

The highest proportion of results reporting the deterioration of the carcass characteristics was identified in the subtopic groups with: feed restriction (n = 25), ingredient substitutions (n = 19), organic production (n = 4), feed alternatives (n = 69), and plant extracts (n = 86). All the investigated carcass characteristics: the carcass and body weight, dressing parentage, breast yield, and abdominal fat were negatively affected by the restriction except for the leg yield, which increased with increasing the restriction level (Englmaierová et al., 2021). Lowering dietary CP levels from 22.5% to 16.5%, had a negative effect on the breast weight and fat pad weight (Adrizal et al., 2019). The replacement of soybean meal with full-fat black soldier fly (*Hermetia illucens l.*) larvae meal in the diets exceeding 50% significantly compromised the carcass, which contained less meat and more abdominal fat (Murawska et al., 2021). The carcass yield of the broilers fed cassava copra meal-based and with 10%- flaxseed diets was poorer compared to the birds fed the control commercial feed (Diarra et al., 2015; Mir et al., 2017b). Increasing the levels of grass-clover protein extract (even up to 24%) reduced feed intake, growth, slaughter weight, and carcass yield (Stødkilde et al., 2020). The chickens fed a diet containing 10% camelia (*Camelina sativa L. Crantz*) seed significantly decreased the proportion of abdominal fat in carcasses (Orczewska-Dudek and Pietras, 2019). Palm fermented kernel cake, corn distillers dried grains, dry residue of cassava and addition of olive leaves affected the carcass negatively (Shafey et al., 2013; Alshelmani et al., 2017; Almeida et al., 2020; Kim et al., 2021).

Some dietary subgroups: organic production (n = 4) feed restriction (n = 25), vitamins (n = 12) and feed alternatives (n = 69) did not induce any effects on the carcass characteristics. Eleroglu et al. (2014) did not report any results for the dry oregano or lemon balm leaves. In 2020 Hussein et al. (2020) did not record any differences in treatment groups with low metabolizable energy diets. Fatemi et al. (2021), did not observe any positive effects of the addition of vitamin D3, neither did Jaapar et al. (2020) for prilled palm or Lan at al. (2020) for sodium butyrate supplementation.

### Enrichment and Carcass Characteristics

Perches and outdoor access have diverse effects on the carcass characteristics. Studies examining these aspects are rare and only four publications in the past ten years examined the effect of perches. The authors found no effect of the provision of perches on the body weight or the carcass yield (Bench et al., 2016; Kiyma et al., 2016; Aksit et al., 2017).

Outdoor access is another possibility to provide broilers with environmental enrichment. The access to an outdoor area was linked to a negative or no effect on the carcass yield in the majority of the studies. The negative effect of providing outdoor access was mentioned by Stadig et al. (2016), Funaro et al. (2014), Skrivanova et al. (2017), and Skomorucha et al. (2020), while Chen et al. (2013), Zaid et al. (2020), Evaris et al. (2021), and Sampels et al. (2021) did not observe any effect of providing outdoor access on the carcass yield. Only Mosca et al. (2018) observed a high general carcass yield in outdoor chickens.

### Stocking Density and the Carcass Characteristics

Various studies reported an effect of stocking density on the carcass and muscle yield. Higher carcass and breast muscle yields were reported for a stocking density between 10 and 15 birds per m^2^ compared to a stocking density between 16 and 20 birds per m^2^, which we consider as low and medium stocking density vs. high stocking density in Cengiz et al. (2015), Henrique et al. (2017), Gholami et al. (2020a), and Nasr et al. (2021). Gholami et al. (2020a) and Costa et al. (2021) compared 10 vs. 15 birds/m^2^ and 8.9 vs. 11.2 birds/m^2^, which both are low stocking density vs. medium stocking density (Costa et al., 2021). They reported a higher muscle yield for lower stocking density. On the contrary, lower carcass and thigh muscle yields were reported for lower stocking density (10-18 birds/m^2^) compared to higher stocking density (20 birds/m^2^) in other studies (Cengiz et al., 2015; Gholami et al., 2020b; Nasr et al., 2021). Furthermore, Tong et al. (2012) reported a higher yield for medium stocking density (17.5 birds/m^2^) than for low and high stocking density (12.5 and 22.5 birds/m^2^).

### Genetics and the Carcass Characteristics

Supplementary material 2 refers to the studies that described the effects of the genetic makeup on broiler carcass characteristics.

Fourteen out of 19 available papers reported significant results. Fast-growing broilers showed the highest carcass yields (3 studies), thigh muscle yield (one study) and breast muscle yields (5 studies) as compared to other broiler types. The highest carcass yields, thigh and drumstick yields were reported for medium-growing broilers (Cömert et al., 2016; Tůmová et al., 2021), as compared to other broiler types. Comparing one breed of slow-growing broilers to a local breed and their crosses, the slow-growing broiler showed higher carcass yields (Cassandro et al., 2015). Growth performance was inconsistent between sexes of slow-growing broilers, with females showing the lowest values and males showing the highest, compared to a fast and a medium-growing line (Tůmová et al., 2021). Higher values were seen for carcass yields of dual-purpose chicken compared to a layer line (Siekmann and Krischek, 2019a). Compared to a fast-growing broiler line higher values of thigh muscle yields were observed (Siekmann et al., 2018a). Similar observations were made in comparison to layer lines (Siekmann and Krischek, 2019b).

## DISCUSSION

For some decades intensive broiler production research has emphasized improved growth performance but now there are increased efforts to improve meat quality (Mir et al., 2017a). The current review examined the links between key husbandry factors and broiler meat quality and carcass characteristics.

Based on the identified papers over the last 10 years, we identified diet as the most researched husbandry factor influencing meat quality and carcass characteristics. Although the results were contentious, the published papers could be categorized into fourteen subtopics reflecting potential areas for more extensive evaluation of dietary effects.

Surprisingly, in a relatively large number of reviewed studies, which investigated dietary treatments, their effects on the carcass or meat quality were negative thus reducing birds' performance, carcass characteristics or meat quality. Negative effects on all three aspects of meat quality (sensory, physical, and chemical), were reported in the studies applying diet restrictions, antibiotic alternatives, plant extracts, protein and feed alternatives. Moreover, both the physical and chemical parameters of the meat were negatively affected by feed form, minerals and microelements, and ingredient substitution. Moreover, supplementation of some vitamins, like for instance application of high concentrations of vitamin C increased lipid oxidation and deteriorated meat quality (Mohiti-Asli and Ghanaatparast-Rashti, 2017).

Even though feed restrictions may be used to control the occurrence of myopathies in broiler chickens (Kuttappan et al., 2013; Mazzoni et al., 2015; Trocino et al., 2015) the reviewed studies most often identified negative effects of the feed restriction both on the carcass and meat quality, mainly on the color parameters, meat pH or reduced bioactive compounds levels (i.e., alpha- and gamma-tocopherol).

On the contrary, evidence from other studies proved minerals and microelements, probiotics and prebiotics, enzymes, organic diets and feeding pasture/roughage to have favorable effects on both meat quality and carcass characteristics. Such dietary strategies may be implemented to alleviate some intensive management strategies negatively affecting birds’ health and welfare, that is, high stocking densities or large group sizes (Estevez and Christman, 2006). Bioactive components found in these types of diet appear to serve as antioxidant, antibacterial and immunomodulatory agents and thus improve the intestinal microflora and morphology of broilers, as well as their physiological conditions, stress responses, antioxidative status, and litter quality (Sugiharto and Ranjitkar, 2019). The supplementation of broiler diets with probiotics improved the carcass yield and meat quality, triggered by proteome alterations, especially the glycolytic proteins (Zheng et al., 2014) but also alleviated stress in broilers (Sugiharto et al., 2017).

Against the expectations, the number of studies investigating the effect of the diet on the carcass and meat quality under an organic production system (considered a very extensive chickens rearing system) was low, in contrast to the growing popularity of this type of production system, especially in Europe. The organic poultry sector has been rapidly growing in certain parts of the EU. Optimizing organic feed formulations with respect to the animals' amino acid requirement, without feeding a surplus of protein, is a challenge, as supplementation with free amino acids is not allowed in the organic production system (Burley et al., 2016). Moreover, the permission to include 5% non-organic feed in organic feed formulations, used mainly for balancing the amino acid composition of the feed, will be phased out in the EU. This will cause an increased need for alternative protein sources with the desired amino acid composition for optimal poultry growth (Van Krimpen et al., 2016). Therefore, it is necessary to examine further what is the effect of organic production systems and novel dietary organic components on the chicken carcass and meat quality.

In extensive rearing systems, including organic, but also, in some intermediate ones, pasture or roughage make important components of a bird's diet. Pastures may constitute a source of energy and proteins for chickens providing a range of bioactive compounds such as antioxidants, hypocholesterolemic, and anticarcinogenic compounds available (Ponte et al., 2008). Previously, Ponte et al. (2008) reported that pasture intake was 2.5% (4 g dry matter (DM) per chick daily); Lorenz et al. (2013) observed that grazing can account for 10 to 15% of the feed intake (2–5 g DM/chick/day) (Lorenz et al., 2013). Nevertheless, the rearing system with outdoor access had a relatively minor effect on chicken breast meat quality in reviewed studies. The results also indicated that meat quality in experiments with free-range housing can be affected by a fat source in the basal diet.

Poultry meat production efficiency depends on diet composition, as one of the main factors (Semjon et al., 2020). The current literature review revealed a large interest in the potential use of new and sustainable feedstuff for broilers. We have observed a clear tendency, especially in the most recent studies to move toward alternative, unconventional and local feed ingredients or nutritional strategies (Sugiharto and Ranjitkar, 2019), which can be considered diet extensification. This can be caused, among others, by the urge to use by-products from other food and feed production in light of the increased global feed prices (Sugiharto and Ranjitkar, 2019), or the need to reduce negative effects on the environment. Nevertheless, in many cases challenges related to such feed components were described. While for instance flaxseed is potentially useful with its high α-linolenic acid, cassava, manioc, tapioca (*Manihot esculenta*) roots and copra meal were described to have limited food or industrial uses (Konieczka et al., 2017). Consequently, the results of the reviewed studies showed that unconventional ingredients frequently have negative effects, especially on the carcass characteristics. In some cases, the addition of enzymes or preprocessing of an ingredient can be a solution for broiler producers who consider the use of alternative feed sources. The reviewed studies proved that enzymes used when feeding for instance, broken rice, palm kernel cake, or distillers grains improved selected carcass characteristics.

Some reviewed research papers presented results related to either carcass characteristic or meat quality achieved by the application of commercial preparations, for instance enzyme complexes or feed where the full formulation was not revealed for the commercial reasons. Similarly, herbal mixtures prepared in proportions for the purpose of a particular study were incomparable with other studies even if the key compound, for instance of plant origin, was the same.

Providing access to environmental enrichment is increasingly demanded by several label programs. Environmental access could include the provision of perches or pecking stones or the access to an outdoor area. In this review furnished cages were not regarded as environmental enrichment, nor was litter, as it is demanded by law in most European countries. Indoor or outdoor environmental enrichment is assumed to bring about higher activity of birds, which affected the meat quality and carcass composition in a positive or negative way.

The chemical meat quality (pH, content of lipids, fat, protein, or moisture) was not affected or analyzed by the studies regarding the indoor environment such as perches or light. Access to an outdoor area influenced the chemical meat quality, however the results of different studies varied and showed no clear tendency affecting protein and fat content, moisture, or pH in meat. Regarding the lipid composition, the majority of the studies observed a positive influence of the outdoor access (Chen et al., 2013; Funaro et al., 2014; Stadig et al., 2016; Michalczuk et al., 2017; Evaris et al., 2021; Sampels et al., 2021).

Studies of an effect of indoor enrichment on physical meat quality such as WHC, cooking loss, drip loss, thawing loss, shear force, or white striping were rare. No conclusion on the direction of one of the intrinsic factors can be drawn, but the outdoor access influenced the physical meat quality in various ways. However, similar to the chemical meat quality, the studies had inconsistent results and only a clear effect of higher shear force was observed, explained as the outcome of increased broiler activity (da Silva et al., 2017; Michalczuk et al., 2017).

In general, the color is more favorable if L* is lower and the a* and b* values are higher (Kirkpinar et al., 2001). Some authors providing perches as indoor enrichment stated that the color of the meat was influenced and a* and b* values which were higher due to increased activity which led to more myoglobin stored in the muscle (Aksit et al., 2017). Conversely, other studies observed a decrease of a* and b* (Kiyma et al., 2016), indicating that more research is needed to quantify the effect.

The majority of studies observed an increase in L* upon the provision of outdoor access. Considering b*, an increase or no effect was observed by the authors, assuming that if a change in color is observed with outdoor access, the meat was more yellow. Interestingly, the majority of studies (5 out of 6) did not see an influence of outdoor access on the a* of the meat (Stadig et al., 2016; Michalczuk et al., 2017; Skomorucha et al., 2020; Zaid et al., 2020; Sampels et al., 2021), indicating that the additional activity does not have a negative influence. One study reported that outdoor birds showed a darker and more yellow color of breast meat compared with those reared without outdoor access (Michiels et al., 2014).

The carcass characteristics or specifically carcass yield, breast, thigh, or drumstick yield were not influenced by the provision of perches. Outdoor access in contrast was mentioned by most studies to lead to a lower yield or performance. Stadig et al. (2016) proposed, for example, that free range negatively affected body weight, but had positive effects on meat quality, taste, and composition. Other authors conclude that outdoor access had no effect on the performance and carcass yield but improved the meat quality (Chen et al., 2013).

Our analysis of the presented studies showed that environmental enrichment such as perches or free range can influence meat quality and the carcass characteristics. However, other factors, related to management and husbandry procedures may influence the responses.

Effect of stocking density was identified on both meat quality and carcass characteristics. The higher meat pH found by Nasr et al. (2021) for 18 birds/m^2^ than for 20 birds/m^2^ may not say very much, since both stocking densities are considered as high. However, higher meat pH was found in most studies for a low stocking density. Low meat pH is associated with lower meat quality due to decreased glycogen deposits, cooking loss and protein denaturation (Castellini et al., 2002; Jeong et al., 2020; Nasr et al., 2021). Wu et al. (2020b) indicated that high stocking density led to up-regulation of glycolysis and fat metabolism, which can increase meat pH decline after death. This means that the meat of lower stocking densities was of better quality. A higher fat content for lower stocking densities may be due to higher feed intake for lower stocking densities. As this was only reported by one paper, it requires further investigation. Lower cooking loss and drip loss for lower stocking density may indicate that high stocking density may decrease WHC. This could be due to oxidative stress, which may cause reduced muscle strength and function (Wu et al., 2020a,b). The result of Goo et al. (2019) of a lower WHC for lower stocking density is in contrast with the other studies and provides a negative effect. However, this lower WHC was noted by only one paper, while more papers indicated higher WHC. So, overall, it can be assumed that WHC is better at lower stocking densities. Skřivanová et al. (2017) reported that shear force is lower for free range than for indoor housed chickens, and this could be associated with the fact that muscle fiber characteristics were affected by the stocking density. However, only one study reported this effect, and this study compares indoor with outdoor systems. This may not be a valid comparison, so it merits further study. The contrasting results of Goo et al. (2019) and Wu et al. (2020a) about meat color may not be comparable, since the compared stocking densities are different. Goo et al. (2019) indicated that results may be inconsistent due to varying experimental factors. Wu et al. (2020a) indicated that high L* is often associated with muscle diseases and can be caused by heat or oxidative stress. The lower acceptability of meat from chickens raised at lower stocking densities may be influenced by the deteriorated muscle fiber characteristics in meat from birds reared at low stocking densities (Skřivanová et al., 2017; Ebeid et al., 2019). Chemical, physical, and sensory meat parameters are interrelated, and different effects of stocking density have been reported, thus, the effect of stocking density on these meat quality aspects is yet unclear and merits further study.

Higher carcass and breast muscle yields for lower stocking densities may be due to a decreased accessibility to feeders and drinkers at high stocking densities and thus a decreased feed intake (Henrique et al., 2017; Gholami et al., 2020a). Furthermore, climate conditions may also be of influence. Henrique et al. (2017) reported that birds reared at high stocking densities in hot climates needed part of their energy to maintain their body temperatures, which leaves less energy to invest in carcass and muscle yields compared to lower stocking densities.

Overall, it seems that low stocking densities may have a positive effect on meat quality. But there is not much literature showing significant effects. Furthermore, many papers compare different stocking densities, which makes a viable comparison difficult. Therefore, further research is recommended to form/draw a uniform conclusion.

The results in this literature review indicated that the extensification of the genetic aspects of the broiler husbandry, such as the use of slow-growing or dual-purpose birds, does not always have a clearly beneficial impact on meat quality parameters and carcass characteristics. On the other hand, the intensive developments in fast-growing broilers traits caused muscle abnormalities and defects and, consequently, impaired meat quality (Petracci et al., 2015).

More studies focused on meat quality aspects than carcass characteristics and the majority of studies focused on different strains of fast-growing broilers. More studies compared the meat quality parameters and carcass characteristics of various broiler breeds or hybrids of one type and not across breeds or hybrids with varying rates of growth. What followed was that, for instance, the carcass characteristics parameters in slow-growing broilers could only be compared between studies or with local breeds. For dual-purpose breeds, only 2 papers reported positive effects on drumstick yields compared to layer lines and fast-growing broilers (Siekmann et al., 2018a; Siekmann and Krischek, 2019a) and only one compared positive effects for the carcass yields to results in layer lines (Siekmann and Krischek, 2019b). This hindered our design to link different levels of genetic extensification to chicken meat quality and carcass characteristics, across various extensification levels typical of certain production systems. Moreover, the applied definitions of fast-, medium-, slow-growing lines and dual-purpose lines were inconsistent, and no clear boundaries were set other than those in the description of particular genotypes defined by the genetic companies.

Environmental and management factors contribute to between 65% and 90% of the breast meat quality parameters (Bailey et al., 2015). Such propositions make it possible to obtain genotypes with higher performance for local environmental conditions (Vayego et al., 2008). Interactions between the husbandry factors were rarely considered in the reviewed studies. For instance, the low meat pH was linked to lower meat quality (Jeong et al., 2020; Nasr et al., 2021). Thus, as we indicated, higher meat pH found in fast-growing broilers, as compared to slow-growing and dual-purpose birds, may mean better quality of meat. However, these differences are often influenced by other factors, like the age of the birds at the processing. On the other hand, slower-growing broilers are typically raised at lower stocking densities than conventional strains and oftentimes in alternative organic or pasture-based systems. Consequently, their meat quality was expected to be better, which has not been confirmed by the current findings.

As the gastrointestinal tract is being extensively modified by the selection process, changes in the broiler digestive capacity have been observed. It may favor protein deposition and reduce fat accumulation (De Verdal et al., 2011). The low number of studies concerning fat and lipids levels in broiler meat depending on their genetic background still does not allow for a conclusion about the effect of the genotype on those parameters.

Current results indicated that the levels of parameter L* for meat color may be influenced more by the genotype and less by belonging to particular growth rate genetic groups. Previously, slow-growing broilers with access to the outdoors showed yellower breast meat compared with the redder breast meat of slow-growing birds raised indoors, yet this effect was not observed in the conventional birds. Consequently, it is important to interpret the identified results from a broad perspective of the environmental conditions and the animal's response to the environment which can affect meat quality. Another important aspect for consideration when interpreting the current findings is that although some of the research was conducted comparing the meat characteristics of slow-growing and conventional broilers, the strains of birds and housing systems used vary greatly by geographical region.

The current literature review did not allow us to make conclusive statements about genotype effect on WHC, drip-, thaw-, or cooking losses and shear forces.

We identified a relatively low number of studies concerning carcass characteristics for different genetic types (6 for fast-growing broilers, one for medium-growing boilers, 4 for slow-growing boilers, 3 papers for dual-purpose lines and 5 papers with no effect for genetics), which may suggest the need for further studies or reference to prior research. However, for the role genetics has the older findings may no longer be relevant due to the dynamic developments in the field. Overall, carcass characteristics in fast-growing birds were reported to be better, as compared to other growth rate broiler groups. Besides the differences in growth and body weight, genetic selection of fast-growing broiler lines had already been earlier associated with changes in body composition and processing traits. Conventional broiler strains produce greater carcass and breast yields in comparison to unselected strains (Havenstein et al., 1994). The intensive selection for high breast yield driven by the demand for processed products, the preference for breast meat in Western markets (Petracci et al., 2015) and the urge to avoid economic losses brought about muscle disorders including wooden breast and white striping, which are 2 major myopathies reported for conventional strains of broiler chickens (Kuttappan et al., 2013; Petracci et al., 2015). These disorders have posed a growing concern for producers and retailers due to their high incidence and significant economic impacts to the poultry industry (Kuttappan et al., 2013; Petracci et al., 2019).

With the detrimental impacts of the fast growth on the welfare of today's conventional broiler chicken at the forefront of public concern (Meluzzi and Sirri, 2009; Broom, 2017), producers of slower-growing broilers can capitalize on marketing their chicken meat as a higher welfare product. The benefits of higher welfare and better health could also be assigned to medium-growing broilers in intermediate types of rearing systems, popular in some developed EU countries, for instance in the Netherlands (de Jong et al.). The economy of the production of such broiler types is still better than in slow-growing birds. Since, in this review only one study concerned the middle-growing birds, there is an opportunity for further investigations of how to obtain most efficiently valuable meat products from birds which are less susceptible to health issues and better adapted to more extensive rearing systems.

Overall, current literature reviewed revealed that in the less standardized intermediate and extensive broiler production systems in which the variability of husbandry conditions is very large and using a more extensive pool of breeds and hybrids, remains very limited with regard to meat quality and carcass characteristics.

### Considerations and Limitations

Methodological limitations of the current study were mainly caused by the identified broad body of literature and varied approaches applied. Both the meat quality and carcass characteristics have multifactorial background. Each key husbandry factor can be characterized by many types and levels of each treatment producing different outcomes. Sometimes, the levels of a factor can be described on a continuous scale. This was the case with stocking density or some dietary aspects (i.e., protein or feed restriction levels). Conversely, genetics or enrichment treatments are often unique, which made drawing conclusions a complex endeavor. Moreover, some studies presented results of the interactions between key husbandry factors selected for this review. Reporting of the interaction results was too broad for one publication but might be addressed in a subsequent paper. All studies were set in experimental conditions.

This review also aimed initially to disentangle the confounding interactions between the production system and extensification of genotype, nutrition and quantity and quality of space, so that each factor could be studied independently and applied separately to different systems. This objective was hindered, since the treatment combinations in the reviewed literature were not repeatable, especially so for stocking density and enrichment, where the number of studies described in the current study was relatively low. It is also important to note that next to the search criteria we have listed above, we refrained from the systematic quality control of the studies included. The aim was to provide an overview of completed scientific work and define patterns in the evidence provided. We do not claim to have finally proven the validity or invalidity of any of the potentially key husbandry factors, nor did we assess statistical power and effect sizes. This would have been a tremendous undertaking, as varied indicators and measures with different scales had been used and often relevant information is missing in the papers. Moreover, the different methods of assessing either meat quality or carcass characteristics might have yielded different results. Finally, presented results could be affected by the publication bias, as much of the research is only published if effects are found.

## CONCLUSION

This review provided an overview of links between key broiler husbandry factors: diet, genotype, quantity and quality of space with chicken meat quality and carcass characteristics, across various production systems. Against the expectations, extensification factors, including genetics were relatively poorly studied in relation to meat quality, except diet effects. This was partly the reason as to why the compilation of the results concerning interactions effects between key husbandry factors was not possible within this study. The reviewed studies provided indications that slower growing breeds, enrichment (especially outdoor ranges), lower stocking density and some nutritional aspects can improve meat quality and thus better meet the needs of the consumer. There is a special need for research on those parameters less directly related to economic results, mainly related to meat quality, as those may add value to the product if transparently presented to the consumers.

## Appendix Supplementary materials

Image, application 1

Image, application 2

## Footnotes

Supplementary material associated with this article can be found in the online version at doi:10.1016/j.psj.2022.102384.
