# Supplementary material for: Linking key husbandry factors to the intrinsic quality of broiler meat
Source: Poult Sci. 2022 Dec 6;102(2):102384. doi: 10.1016/j.psj.2022.102384 (PMC9801217; doi:10.1016/j.psj.2022.102384)
Supplement: Supplementary file 1 [file mmc1.docx]

Keywords query strings used in the literature search for each key husbandry factor: diet, stocking density, genetics and enrichment were composed of 3 parts. Two parts of each of the keywords strings were equal for all the key husbandry factors: keywords related to various aspects of meat quality and carcass characteristics (A) and keywords related to the broilers (B). Third part of the keywords string was unique for each key husbandry factor (C). Titles (TI) of the articles only were searched for the keywords query strings.

Part A of the keywords string (equal for all four key husbandry factors):

*TI=(Meat OR Carcass) AND TI=(Quality OR Characteristics OR Conformation OR Sensory OR Nutritional OR Physical OR Composition OR Weight OR Yield OR Lean Percentage OR Percentage Of Primals OR Length OR Bruises OR Damage OR Trim Loss OR Woody Breast OR WB OR Spaghetti Meat OR SM OR White Striping OR WS OR Deep Pectoral Myopathy OR DPM OR Immature Intramuscular Connective Tissue Disorder OR IMCT OR Myopathy OR Myopathies OR Aroma OR Taste OR Flavour OR Flavor OR Texture OR Colour OR Color OR Fatty Acids OR Mineral OR Minerals OR Vitamin OR Vitamins OR Amino Acids OR Drip Loss OR Cooking Loss OR Water Holding Capacity OR WHC OR Tenderness OR Marbling OR IMF OR Ph OR Moisture OR Protein OR Fat OR Ash OR Volatile OR Volatiles)*

Part B of the keywords string (equal for all four key husbandry factors):

*TI= (Poultry OR Broiler OR Chicken OR Dual-Purpose OR Layer)*

Part C of the keywords string (unique for each of the four key husbandry factors):

Diet:

*TI= (Diet OR Nutrition OR Feed OR Energy OR Protein)*

Stocking density:

*TI= (Stocking density OR Space allowance)*

Genetics:

*TI= (Genetics OR Genotype OR Genetic constitution OR Line OR Lineage OR Genes OR Genetic makeup OR Genetic make-up OR Biology OR Heredity OR Genome OR Hubbard OR Hubbard ISA JA 757 OR Hubbard ISA JA 787 OR Lohmann OR Lohmann Brown OR male Lohmann brown OR Lohmann Sandy OR Les Bleues OR Bresse chicken OR Ranger OR Red Ranger chicken OR Dual-Purpose OR Ranger Classic OR Rowan Ranger OR Male Chicks OR Slow growing OR Male Layer OR Dual-purpose breed OR Aviagen OR Aviagen Ranger Savanna OR Ranger Savanna OR Rowan Range OR Hubbard slow growth males OR Hubbard slow growth OR Hubbard JA 57 OR Hubbard JA 87)*

Environmental enrichment:

*TI= (Environmental Enrichment OR environment OR Enrichment OR Straw bales OR Elevated platforms OR Platform OR Perch OR Laser OR Enhancement OR Human OR Free range OR Outdoor OR Panels OR Barriers OR Veranda OR Wintergarden OR Winter garden OR Pecking stones OR Pecking OR Pipes)*
