# Supplementary material for: Linking key husbandry factors to the intrinsic quality of broiler meat
Source: Poult Sci. 2022 Dec 6;102(2):102384. doi: 10.1016/j.psj.2022.102384 (PMC9801217; doi:10.1016/j.psj.2022.102384)
Supplement: Supplementary file 2 [file mmc2.docx]

| Parameters | | Broiler genetics, which in the referenced scientific article was identified superior with regard to a certain parameter, as compared to the other investigated broiler genetics^1^ | | | | | | No significant effect identified |
| --- | --- | --- | --- | --- | --- | --- | --- | --- |
|  |  | *Growth rate* | | | *Dual purpose breeds* | *Layer lines* | *Hybrids, local and other breeds* |  |
|  |  | *Fast* | *Intermediate* | *Slow* |  |  |  |  |
| **Meat quality** | |  |  |  |  |  |  |  |
| Chemical quality | pH | Aksoy et al., 2021  Chadová et al., 2021  Narinç et al., 2015  Siekmann et al., 2018a  Wen et al., 2017 | x^2^ | Koçer et al., 2018  Samples et al., 2021^+^  Tasoniero et al., 2018^+^ | x | x | Cassandro et al., 2015*  Jlali et al., 2012 | Cömert et al., 2016  Hassan et al., 2021^+^  Kralik et al., 2015  Michalcuk et al., 2016  Özbek et al., 2020  Sarica et al., 2014  Siekmann et al., 2018b  Sosnówka-Czajka et al., 2017^+^ |
|  | moisture | x | x | x | x | x | x | Michalcuk et al., 2016  Özbek et al., 2020  Sarica et al., 2014 |
|  | protein | Koçer et al., 2018 | x | Chodová et al., 2021  Dalle Zotte et al., 2020  Devetkal et al., 2019  Pietrzak et al., 2013 | x | x | x | Cömert et al. 2016  Michalcuk et al., 2016  Özbek et al. 2020  Sarica et al., 2014  Sosnówka-Czajka et al., 2017^+^ |
|  | fat | Pietrzak et al., 2013  Sosnówka-Czajka et al., 2017^+^ | Boschetti et al., 2016 | Boschetti et al., 2016  Dal Bosco et al., 2012  Dalle Zotte et al., 2019^+^  Sosnówka-Czajka et al., 2017^+^ | Mueller et al., 2020 | Englmaierová et al., 2020^+^ | Paredes and Vásquez, 2020 | Cömert et al,. 2016  Dalle Zotte et al., 2020  Michalcuk et al,. 2016  Özbek et al., 2020  Popova et al., 2016^+^  Sarica et al., 2014  Sosnówka-Czajka et al., 2017^+^ |
|  | lipids | x | x | Dalle Zotte et al., 2019^+^ | x | x | x | Cömert et al., 2016  Dalle Zotte et al., 2020  Michalcuk et al., 2016  Özbek et al., 2020  Popova et al., 2016+  Sarica et al., 2014  Sosnówka-Czajka et al., 2017^+^ |
| Physical quality | WHC | Barbosa et al., 2017^+^  Hassan et al., 2021^+^  Pietrzak et al., 2013 | x | Koçer et al., 2018 | Mueller et al., 2020  Siekmann and Krischek, 2019a | x | x | Łukasiewicz et al., 2015  Sarica et al., 2014  Sosnówka-Czajka et al., 2017^+^ |
|  | cooking loss | Barbosa et al., 2017^+^  Chodová et al., 2021  Hassan et al., 2021+  Koçer et al., 2018  Siekmann et al., 2018a  Wen et al., 2017 | Chodová et al., 2021 | Pietrzak et al., 2013 | x | x | Cassandro et al., 2015* | Aksoy et al., 2021  Kralik et al., 2015^+^  Mueller et al., 2018  Paredes and Vásquez, 2020  Siekmann et al., 2018b |
|  | drip loss | Chodová et al., 2021  Koçer et al., 2018 | Chodová et al., 2021 | Singh et al., 2021  Wen et al., 2017 | Siekmann et al., 2018a | x | Jlali et al., 2012 | Kralik et al., 2015^+^  Paredes and Vásquez, 2020  Siekmann et al., 2018b |
|  | thawing loss | Chodová et al., 2021 | Almasi et al., 2015  Chodová et al., 2021 | Mueller et al., 2018 | Mueller et al., 2018  Siekmann et al., 2018a | x | Cassandro et al., 2015* | Aksoy et al., 2021  Siekmann et al., 2018b  Sosnówka-Czajka et al., 2017^+^ |
|  | shear force | Barbosa et al., 2017+  Mueller et al., 2018 | x | Chodová et al., 2021  Devetkal et al., 2019  Samples et al., 2021+ | Mueller et al., 2020 | Englmaierová et al., 2020+ | x | Drobnyak et al., 2019  Kralik et al., 2015^+^  Özbek et al., 2020  Siekmann et al,. 2018b  Siekmann and Krischek, 2019b |
|  | white striping | Dalle Zotte et al., 2020 (6) | x | x | x | x | x | x |
| Sensory quality | appearance | x | x | x | x | x | x | Michalcuk et al., 2018 |
|  | color | x | x | x | x | x | x | Drobnyak et al., 2019  Kralik et al., 2015^+^  Mueller et al., 2018  Sarica et al., 2014  Siekmann et al,. 2018b  Siekmann and Krischek, 2019b  Sosnówka-Czajka et al., 2017^+^ |
|  | L* | Barbosa et al., 2017+  Batkowska et al., 2015  Chodová et al., 2021  Da Costa et al., 2017^+^  Hassan et al., 2021^+^  Singh et al., 2021  Wen et al., 2017 | Almasi et al., 2015  Chodová et al., 2021 | Aksoy et al., 2021  Almasi et al., 2015  Canoğulları et al., 2019  Eleroğlu et al., 2013^+^  Narinç et al., 2015  Tasoniero et al., 2018^+^  Wen et al., 2017 | x | x | Cassandro et al., 2015*  Jlali et al., 2012 | Özbek et al., 2020 |
|  | b* | Barbosa et al., 2017^+^ | x | x | x | x | Cassandro et al., 2015* | Dalle Zotte et al., 2020  Sarica et al., 2014  Michalczuk et al., 2016  Popova et al., 2016^+^  Cömert et al., 2016  Özbek et al., 2020  Sosnówka-Czajka et al., 2017^+^ |
|  | a* | Barbosa et al., 2017^+^  Hassan et al., 2021^+^ | x | Eleroğlu et al., 2013^+^  Narinç et al., 2015  Özbek et al., 2020  Singh et al., 2021  Tasoniero et al., 2018^+^ | x | x | Cassandro et al., 2015* | Aksoy et al., 2021 |
|  | taste | x | x | Pellattiero et al., 2020 | x | x | x | x |
|  | odor | x | x | x | x | x | x | x |
|  | texture | x | x | Pellattiero et al., 2020 | x | x | x | x |
| **Carcass characteristics** | |  |  |  |  |  |  |  |
| carcass yield | | Barbosa et al., 2017^+^  Canoğulları et al., 2019  Cömert et al., 2016  Siekmann et al., 2018a | x | Cassandro et al., 2015*  Eleroğlu et al., 2014^+^  Sosnówka-Czajka et al., 2017^+^ | Siekmann and Krischek, 2019a | Englmaierová et al., 2020^+^ | Almeida et al., 2013  Batkowska et al., 2015 | Ipek and Sozcu, 2017  Jlali et al., 2012  Maharjan et al., 2021^+^  Trocino et al., 2015  Zaglool et al., 2019^+^ |
| breast muscle yield | | Batkowska et al., 2015  Cömert et al., 2016  Siekmann et al., 2018a  Siekmann et al., 2018b | x | Eleroğlu et al., 2014^+^  Samples et al., 2021^+^ | x | Siekmann and Krischek, 2019b | Cassandro et al., 2015* | Jlali et al., 2012 |
| thigh muscle yield | | Siekmann et al., 2018a | Cömert et al., 2016 | Eleroğlu et al., 2014^+^ | Siekmann et al., 2018b  Siekmann and Krischek, 2019b | x | Batkowska et al., 2015 | Jlali et al., 2012 |
| drumstick yield | | x | Cömert et al., 2016 | Eleroğlu et al., 2014^+^ | x | x | Batkowska et al., 2015 | Jlali et al., 2012 |

^1^ Broiler genetics which in the referenced in this table scientific article was identified posterior with regard to a certain parameter has been referred to in the publication text.

^2^ No scientific article found.
